# Effectiveness of Onsite Nurse Mentoring in Improving Quality of Institutional Births in the Primary Health Centres of High Priority Districts of Karnataka, South India: A Cluster Randomized Trial

**DOI:** 10.1371/journal.pone.0161957

**Published:** 2016-09-22

**Authors:** Krishnamurthy Jayanna, Janet Bradley, Prem Mony, Troy Cunningham, Maryann Washington, Swarnarekha Bhat, Suman Rao, Annamma Thomas, Rajaram S, Arin Kar, Swaroop N, Ramesh B M, Mohan H L, Elizabeth Fischer, Maryanne Crockett, James Blanchard, Stephen Moses, Lisa Avery

**Affiliations:** 1 Department of Community Health Sciences, University of Manitoba, Winnipeg, Manitoba, Canada; 2 Karnataka Health Promotion Trust, Bangalore, Karnataka, India; 3 St John’s National Academy of Health Sciences, Bangalore, Karnataka, India; 4 IntraHealth International, Chapel Hill, North Carolina, United States of America; Penang Medical College, MALAYSIA

## Abstract

**Background:**

In India, although the proportion of institutional births is increasing, there are concerns regarding quality of care. We assessed the effectiveness of a nurse-led onsite mentoring program in improving quality of care of institutional births in 24/7 primary health centres (PHCs that are open 24 hours a day, 7 days a week) of two high priority districts in Karnataka state, South India. Primary outcomes were improved facility readiness and provider preparedness in managing institutional births and associated complications during child birth.

**Methods:**

All functional 24/7 PHCs in the two districts were included in the study. We used a parallel, cluster randomized trial design in which 54 of 108 facilities received six onsite mentoring visits, along with an initial training update and specially designed case sheets for providers; the control arm received just the initial training update and the case sheets. Pre- and post-intervention surveys were administered in April-2012 and August-2013 using facility audits, provider interviews and case sheet audits. The provider interviews were administered to all staff nurses available at the PHCs and audits were done of all the filled case sheets during the month prior to data collection. In addition, a cost analysis of the intervention was undertaken.

**Results:**

Between the surveys, we achieved coverage of 100% of facilities and 91.2% of staff nurse interviews. Since the case sheets were newly designed, case-sheet audit data were available only from the end line survey for about 80.2% of all women in the intervention facilities and 57.3% in the control facilities. A higher number of facilities in the intervention arm had all appropriate drugs, equipment and supplies to deal with gestational hypertension (19 vs.3, OR (odds ratio) 9.2, 95% C.I 2.5 to33.6), postpartum haemorrhage (29 vs. 12, OR 3.7, 95% C.I 1.6 to8.3); and obstructed labour (25 vs.9, OR 3.4, 95% CI 1.6 to8.3). The providers in the intervention arm had better knowledge of active management of the third stage of labour (82.4% vs.35.8%, AOR (adjusted odds ratio) 10, 95% C.I 5.5 to 18.2); management of maternal sepsis (73.5% vs. 10.9%, AOR 36.1, 95% C.I 13.6 to 95.9); neonatal resuscitation (48.5% vs.11.7%, AOR 10.7, 95% C.I 4.6 to 25.0) and low birth weight newborn care (58.1% vs. 40.9%, AOR 2.4, 95% C.I 1.2 to 4.7). The case sheet audits revealed that providers in the intervention arm showed greater compliance with the protocols during labour monitoring (77.3% vs. 32.1%, AOR 25.8, 95% C.I 9.6 to 69.4); delivery and immediate post-partum care for mothers (78.6% vs. 31.8%, AOR 22.1, 95% C.I 8.0 to 61.4) and for newborns (73.9% vs. 32.8%, AOR 24.1, 95% C.I 8.1 to 72.0). The cost analysis showed that the intervention cost an additional $5.60 overall per delivery.

**Conclusions:**

The mentoring program successfully improved provider preparedness and facility readiness to deal with institutional births and associated complications. It is feasible to improve the quality of institutional births at a large operational scale, without substantial incremental costs.

**Trial Registration:**

ClinicalTrials.gov NCT02004912

## Introduction

Maternal and newborn mortality rates in India have declined over the past two decades, but progress remains short of the 2015 targets set out in the millennium development goals (MDG) [1]. Progress has been partly due to a rise in institutional births influenced by the Government of India’s flagship program ‘National Rural Health Mission’ (NRHM), recently re-termed ‘National Health Mission’ (NHM), which adopted a number of innovative approaches and schemes to increase institutional births. Significant investments were also made in strengthening infrastructure, staffing, service delivery and emergency transportation [2–4]. While institutional births have risen over recent years, the quality of care in those institutions has not improved substantially [5,6]. Evidence indicates that the highest number of maternal and newborn deaths occur during delivery and immediately after delivery [7,8]. In India, four maternal complications and three newborn complications contribute to 60% and 80% of total maternal and newborn deaths respectively [9,10]. Adequate focus on quality of care and management of complications, in addition to efforts to improve coverage of institutional births, is needed to achieve desired reductions in mortality and morbidity [11,12].

Attempts within India to implement quality assurance programs in the past have met with limited success [13]. While governments recognize the need to equip facilities with skilled staff and provide resources to improve the quality of care, factors persist that reduce overall quality of care, such as inadequate pre-service training, lack of systems for continuous monitoring and follow up support after training to upgrade staff skills and practices, inadequacies in the supply of essential drugs and equipment, inadequate referral mechanisms, and poor continuity of care [14]. Poor provider preparedness and facility readiness that contribute to gaps in quality of maternal and newborn care is well documented in the region [5]. However, there is a paucity of published evidence documenting approaches to improve care quality that are feasible for large scale application in India. To address this gap, we designed and tested a program to improve the quality of care of institutional births at primary health centres (PHC) using an on-site nurse mentoring model. Since PHCs are the first point of contact for institutional births, and have received lot of attention and investments under the NRHM, the project in consultation with the government decided to focus its quality improvement efforts at the PHCs. In the Indian public health system, primary health centres (PHCs) serve a rural catchment population of 30,000. PHCs that are open 24 hours a day, 7 days a week (“24x7” PHCs) are meant to be staffed by at least one doctor, also known as a medical officer (MO), and 3–5 staff nurses (SNs) and are the focus of NHM facility strengthening efforts. These facilities offer labour and delivery services, including screening, management and referral of maternal and newborn complications to higher-level referral facilities [4,5]. In northern Karnataka, as per the health management information system (HMIS) about 26% of deliveries occur in the 24/7 PHCs and so any quality of care intervention at this level can potentially impact a substantial number of births in the region.

The specific objectives of the study were to assess the effectiveness of onsite nurse mentoring model in improving the quality of institutional births in the PHCs, with a specific focus on facility readiness and provider preparedness for dealing with maternal and newborn complications. In addition, we present the costs incurred in developing and implementing this model.

## Materials and Methods

### Study setting

The study was conducted in two districts of northern Karnataka, a southern Indian state that has a maternal mortality ratio (MMR) of 144 per 100,000 live births and a newborn mortality rate (NMR) of 22 per 1000 live births [15,16]. The wide disparities between northern and southern Karnataka in relation to health and other indicators are well documented [17]. Inadequate coverage of MNH services in northern Karnataka is well documented [5,18,19]. Eight districts in northern Karnataka (out of 30 in the entire state), with a total population of about 16 million, are considered high priority districts, identified by the Government of India in its ‘Call to Action Strategy’ as requiring additional focus to enhance reproductive, maternal, newborn, child and adolescent health (RMNCH+A) outcomes in the state [20]. As part of a technical assistance program for the government of Karnataka (2009–15), several innovations were designed and implemented within the communities, facilities and the health systems in these high priority districts to improve accessibility, quality and coverage of critical MNCH services. The nurse mentoring intervention was designed as a facility (24/7 PHC) level innovation aiming to improve provider knowledge and skills and enhance facility readiness, through a dedicated cadre of nurse mentors. The study was conducted in the districts of Bellary and Gulbarga.

### Trial design

The effectiveness of the onsite mentoring program was evaluated using a parallel, cluster randomized control study design, with the nurse mentor intervention randomized to facilities and the staff therein. The trial incorporated pre- and post-intervention surveys to measure the changes in facility readiness and provider preparedness.

### Participants and sample

Assuming that 10% of staff nurses had correct knowledge (of all three steps of active management of third stage of labour) at the time of baseline, and that the intervention would improve this to at least 20%, setting alpha level error at 0.05 (95% confidence limits), beta level error at 0.2 (80% power) and intra-class correlation at 0.4, the study required an estimated sample of 54 clusters and 162 staff nurses per arm. The two study districts had 109 functional 24/7 PHCs andone facility was under renovation at the time of the study, and so 108 were included in the study, of which 54 were randomly allocated into an intervention arm and 54 into a control arm. Within the facility, all nursing staff available were interviewed in the study. For case sheet audits all, filled case sheets for the month of July 2013 were included in the study.

### Intervention

The onsite mentoring intervention focused on improving systems and staff functioning within each facility. The 54 intervention facilities received six supportive onsite visits by one of nine nurse mentors during the course of one year (a dedicated nurse mentor for 6 facilities).The mentors had a basic qualification in general nurse midwifery (GNM), and were recruited locally. They were given further training for five weeks in topics related to quality improvement approaches and tools, mentoring skills, and clinical topics on obstetric and neonatal care and primary care systems. In addition they received handholding support in the field once every quarter by clinical experts who helped in reviewing and reinforcing the mentoring skills of the mentors. The mentors attended clinical refresher every six months to renew their clinical skills and practices.

The trained mentors visited facilities allocated to them once every two months and each visit lasted for 3 days. During the visits, they trained the staff in using self-assessment checklists to assess gaps in facility readiness, and develop action plans to address gaps that were identified. Clinical mentorship was provided using multiple approaches such as bed side coaching, case demonstrations, use of case vignettes and job-aids. The mentors particularly focused on care during intrapartum and postpartum periods including essential newborn care alongside recognition, management and referral of common complications during these periods. They used a structured teaching plan during each visit, yet were flexible in responding to the emerging needs in the facilities. Case sheet audits and observations of staff practices helped mentors to plan the clinical mentoring sessions with the staff either in one-to-one or one-to-group sessions as appropriate. Each visit started with planning meeting and ended with a debrief meeting with the facility teams that ensured joint planning, follow-up and continuity.

Prior to the mentoring program, a new case sheet was introduced to the staff nurses and medical officers at all 108 facilities (both intervention and control) through a training update of three days and one day duration, respectively. The new case sheets were developed by the project in consultation with the Karnataka government and piloted as a part of the mentoring intervention. The earlier case sheets were open-ended and not specific to maternal and newborn care. They were restructured to function as job-aid in providing step-by-step guidance to staff for managing women during initial assessment, labour monitoring, delivery and postpartum care, and to facilitate diagnosis of maternal and neonatal complications and their pre-referral management. The restructured case sheets consisted of a delivery record and eight separate complication sheets, one for each of the most common complications (maternal: prolonged / obstructed labor, preeclampsia and eclampsia, antepartum hemorrhage, infection/sepsis, premature rupture of membranes, postpartum hemorrhage; newborn: neonatal asphyxia, sepsis and low birth weight / prematurity). Orientation training was provided to refresh the providers’ knowledge and skills in conducting institutional deliveries, and to achieve a common understanding of the new case sheet. This not only helped the staff as a job-aid, but also served as the monitoring and evaluation tool for the intervention. The case sheets were designed to facilitate easy documentation, and to be used as a checklist aid to assist providers in following standards of care for institutional births and management of common complications. In addition, they served as teaching aids for use by the mentors. They also were meant to be useful to medical officers in auditing provider practices and improving quality of care.

### Outcomes

Outcomes were measured at the facility level, and involved all of the staff in each facility. We hypothesized that regular on-site mentoring by a trained nurse mentor, along with an initial training update and the use of case sheets, would improve facility readiness and provider preparedness more than just the provision of the training update and the case sheets, without the nurse mentor intervention. Facility readiness and provider preparedness was studied particularly related to the management of normal labour and delivery and the four most common maternal complications (postpartum haemorrhage, gestational hypertension, obstructed labour, maternal sepsis), and the three most common newborn complications (birth asphyxia, low birth weight and neonatal sepsis). Facility readiness was defined as having basic infrastructure and staffing, drugs, equipment and supplies to deal with normal labour and delivery, as well as the most important complications encountered. Provider preparedness was defined as adequate knowledge and practice in alignment with standard guidelines for prevention, screening and management of complications.

### Randomization and measurements

An independent team involved in monitoring and evaluation (M&E) generated a random allocation sequence and assigned clusters (facilities) into intervention and control arms. Within each facility, all staff available during mentoring visits were covered by the intervention. During the pre- and post-intervention surveys, facility readiness was assessed through standardized facility audits, provider knowledge through standardized interviews with all available nursing staff during the survey, and provider practice through the audit of patient case sheets. Facility audits captured details about infrastructure, staffing, equipment and supplies; interviews and case sheet audits were designed to capture knowledge and documentation practices of the staff about management of institutional births and associated complications. The instruments were prepared by a team of experts in the fields of obstetrics, neonatology, quality improvement, epidemiology, and monitoring & evaluation. The Government of India and World Health Organization (WHO) guidelines informed the design of study tools and indicators for analysis. Expenditures incurred for staff salaries and training, implementation, monitoring and reviews were also measured.

### Training and data collection

Field investigators with a medical background were recruited and trained for five days in topics related to maternal and newborn care, as well as in the use of the study tools. They were given the opportunity to observe mock interviews and to practice interviewing. After this training, the interviewers had 2 days of field practice in a neighbouring district not included in the study. Simultaneous data collection was carried out in both the intervention and control districts over a period of one month in April 2012 and again in August 2013 (12 months after the actual start of the main mentoring intervention). The interviewers were blinded to allocation. For the case sheet review, the same interviewers collected all filled project case sheets for the month of July 2013, when the intervention facilities received their last round of mentoring visits, and these were reviewed by physicians for documentation and accuracy.

### Ethics approval and consent

The Institutional Ethical Review Board of St John’s Medical College and Hospital in Bangalore approved the study on February 10, 2012. Approvals by the state government of Karnataka were also obtained prior to the study. Signed informed consent was obtained from the facility in-charge and the providers who participated in the study. The study was also registered at ClinicalTrials.gov, Identifier: NCT02004912.

### Data analysis

Data analysis was undertaken at facility level to understand facility readiness (availability of critical drugs, equipment and supplies) by type of complication, and at provider level to understand preparedness (levels of knowledge and practice). Differences in facility readiness and provider knowledge between intervention-baseline and intervention-endline, control-baseline and control- endline, and intervention-endline and control-endline were analysed. The case sheet audit findings were compared between the intervention end-line and control-endline. Baseline data were not available for case sheet audit analysis, as the case sheets were introduced as a part of the intervention. Bivariate analysis was conducted for comparing facility readiness. Multilevel multivariate logistic regression analyses were conducted for provider (staff nurse) knowledge and case sheet audit data to account for intracluster correlation (ICC). The multivariate model for the provider analysis controlled for district, duration since basic nursing training, attendance at full SBA training and SBA refresher training, total years of work experience and average monthly delivery volume in the facility. The multivariate model for the case sheet analysis controlled for district, intervention type, average monthly delivery volume in the facility, number of staff nurses in the facility and the number of staff nurses who attended full SBA training. Odds ratios (ORs) and adjusted odd ratios (AORs) were calculated, and 95% confidence intervals were constructed. Stata version 12.0 (StataCorp, College Station, Texas 77845, USA) was used for statistical analysis.

### Cost analysis considerations

Actual expenditures for implementing the program in all eight northern Karnataka districts during the year 2013–14 were considered for the cost analysis. The costs were related to project staff (salaries, travel and per-diem), capital costs (such as computers and technical content development), print material, and events (training workshops, refresher training, and review meetings). The costs were categorized into one-time and recurring costs. One-time costs included expenditures that were made once during the program period, such as initial induction training and capital costs; recurring costs included expenditures that recurred regularly, such as staff salaries, travel, review meetings, etc. The costs were later computed for a single district to understand the cost implications for implementing this model in a typical high priority district in the Indian setting. Calculations were also made to understand how much it cost to improve the quality of a single delivery in a rural institutional setting.

## Results

### Cluster allocation

**Fig 1:**
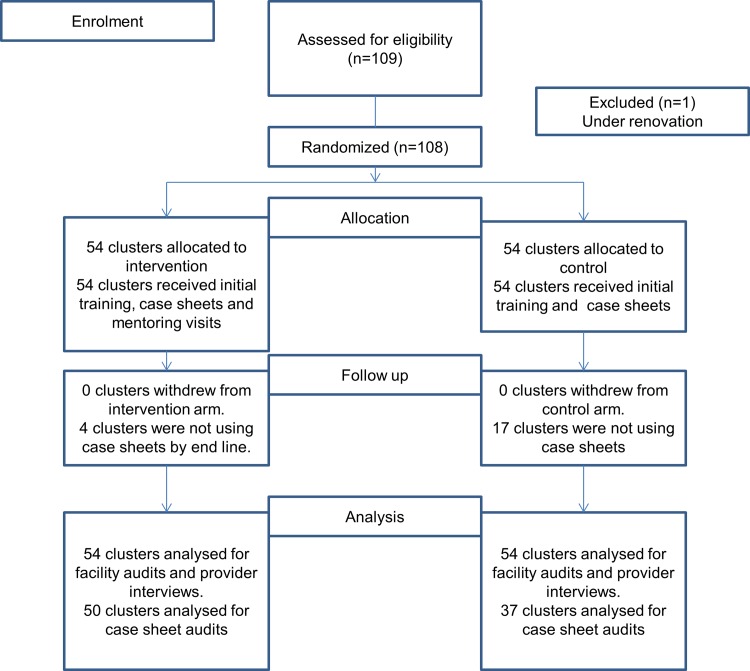
Consort 2010 flow diagram.

**Fig 2:**
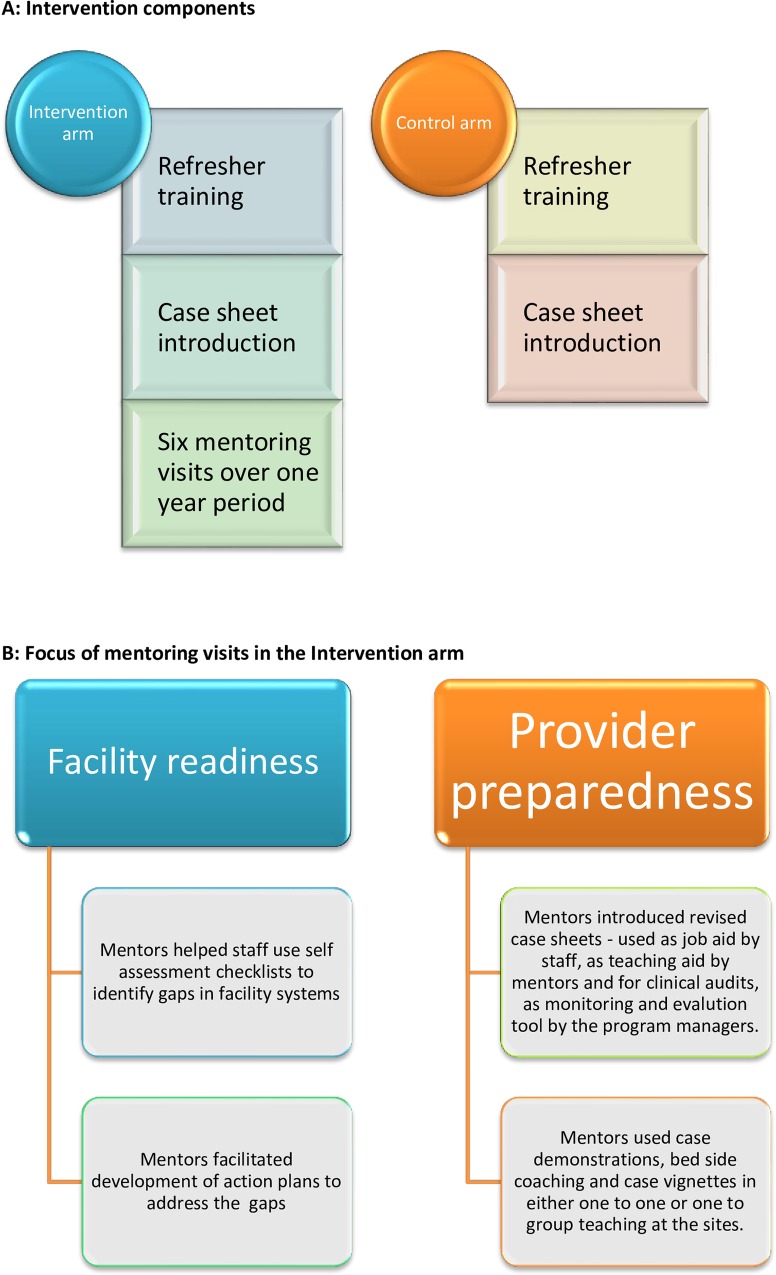
Intervention components in the intervention and control arm.

All assigned (108) clusters (facilities) received the intended intervention over one year period from August 1, 2012 to July 31, 2013. We did not encounter any loss or exclusion of clusters for facility audits and provider interviews. However, as the case sheets were introduced as part of the intervention, we could not establish baseline for these. By the end of one year, 37 control and 50 intervention facilities were using the case sheets and so case sheet data from these facilities were included in the end line analysis. Fig 1 illustrates the allocation of clusters as per the consort flow diagram. Fig 2 demonstrates the differences in the intervention and control arm and highlights the specific components focused in the intervention arm.

### Staffing details

Most facilities had 3 staff nurses available who provided maternal and newborn care. This number dropped marginally during the year in both the intervention and control sites, but the changes were not statistically significant (Table 1). The control arm at baseline had a higher number of staff nurses who had received training in skilled birth (standard module lasting 21 days) and this number increased in both arms. The availability of medical officers was mostly sub-optimal (<1 per facility), except in the control sites at baseline. Their availability dropped in both arms over the course of the year, but these declines were not significant. More medical officers in the control arm of the baseline survey had received training in skilled birth, and this difference was significant (0.8 vs. 0.6, p = 0.041).

**Table 1:** Study coverage and respondent characteristics.

Parameter	Intervention (2012)	Intervention (2013)	Control (2012)	Control (2013)
**Facility staff profile**				
Coverage of sites (#/ %)	54 (100%)	54 (100%)	54 (100%)	54 (100%)
Staff nurses employed (#/mean no per facility) • p^a^ = 0.810 • p^b^ = 0.462 • p^c^ = 0.080 • p^d^ = 0.103	160 (2.96)[SD:0.43]	152 (2.81) [SD:0.44]	161 (2.98) [SD:0.36]	155 (2.87) [SD:0.34]
Staff nurses with skilled birth training (#/mean no per facility) • pa = 0.049 • pb = 0.262 • pc = 0.373 • pd = 0.298	121 (2.24) [SD:0.87]	129 (2.39) [SD:0.86]	137 (2.54) [SD:0.66]	139 (2.57) [SD:0.72]
Medical officers employed (#/mean no per facility) • p^a^ = 0.226 • p^b^ = 0.521 • p^c^ = 0.477 • p^d^ = 0.277	49 (0.91) [SD:0.45]	46 (0.85) [SD:0.36]	54 (1.00) [SD:0.34]	49 (0.91) [SD:0.52]
Medical officers with skilled birth training (#/mean no per facility) • p^a^ = 0.041 • p^b^ = 0.434 • p^c^ = 0.852 • p^d^ = 0.116	32 (0.59) [SD:0.53]	31 (0.57) [SD:0.50]	43 (0.80) [SD:0.49]	35 (0.65) [SD:0.48]
**Interviewed staff nurse profile**				
Coverage of staff nurses (#/%)	147 (92%)	136 (89.5%)	148 (92%)	137 (88.4%)
Surveyed nurses trained in skilled birth (21 day) (#/%) • p^a^ = 0.018 • p^b^ = 0.123 • p^c^ = 0.020 • p^d^ = 0.002	110 (74.8%)	121 (88.9%)ǂ	127 (85.8%)	129 (94.2%)
Mean no of years since skilled birth training of surveyed staff nurses • p^a^ = 0.416 • p^b^ = 0.331 • p^c^ = 0.012 • p^d^ = 0.005	1.97 [SD:1.14]	2.47 [SD:1.47]	2.12 [SD:1.66]	2.67 [SD:1.80]
Surveyed nurses trained in skilled birth refresher training (3 days) training (#/%) • p^a^ = 0.045 • p^b^ = 0.069 • p^c^< 0.001 • p^d^<0.001	46 (31.3%)	81 (59.6%)ǂ	63 (42.6%)*	96 (70.1%)
Mean no of months since skilled birth refresher training of surveyed staff nurses • p^a^ = 0.736 • p^b^ = 0.713 • p^c^< 0.001 • p^d^< 0.001	5.40 [SD:3.02]	13.10 [SD:9.68]	5.20 [SD:3.80]	12.60 [SD:9.18]

p^a^: p value for difference between control and intervention in 2012

p^b^: p value for difference between control and intervention in 2013

p^c^: p value for difference in intervention sites between 2012 and 2013

p^d^: p value for difference in control sites between 2012 and 2013

p values for mean are based on t-test and for percentages are based on chi-square test; SD-standard deviation

### Response rates

We achieved 100% coverage of facilities during the baseline and endline surveys. We were able to interview 92% of staff nurses in both the arms at baseline. This changed to 89% in the intervention arm and 88% in the control arm at endline, and these changes were not statistically significant. The number of respondents who had received training in skilled birth (21 days) as well as a short refresher training (3 days) increased over time in both arms and these changes were significant in both arms.

### Facility readiness

The facility audits examined availability of critical drugs, equipment, supplies and referral systems, as a reflection of the readiness of facilities to deal with institutional births and associated complications (Table 2). We specifically assessed if facilities had all the critical drugs, equipment and supplies to deal with a particular complication. While none of the study facilities had all of the necessary supplies to deal with gestational hypertension in 2012, 19 facilities in the intervention arm and 3 facilities in the control arm attained readiness by the end of the study period (OR 9.2, 95% C.I 2.5 to 33.6). Similarly, intervention facilities showed significant improvements over control facilities in readiness for managing postpartum haemorrhage (29 vs. 12, OR 3.7, 95% C.I 1.6 to 8.3), maternal sepsis (29 vs. 13, OR 3.7, 95% CI 1.6 to 8.3) and obstructed labour (25 vs. 9, OR 4.3, 95% C.I 1.8 to 10.5). Nine intervention facilities had attained readiness to deal with newborn complications at end line vs none at base line; the change was marginal in the control arm (OR 2.5, 95% CI 0.7 to 8.7). More facilities in the intervention arm had comprehensive referral systems than in the control arm (25 vs. 5, OR 8.4, 95% C.I 2.9 to 24.5). The specifics related to drugs, equipment and supplies for each category are detailed in S1 File.

**Table 2:** Facility readiness to deal with maternal and newborn complications in the intervention and control facilities.

Category	Intervention 2012 (#/%) N = 54%)	Intervention 2013 (#/%) N = 54	Intervention 2012 vs.2013 OR, 95% CI and p value	Control 2012 (#/%) N = 54	Control 2013 (#/%) N = 54	Control 2012 vs. 2013 OR, 95% CI and p value	Intervention vs. control, 2013 OR,95% CI and p value
Gestational hypertension	0	19 (35.2)	-	0	3 (5.6)	-	9.2 (2.5–33.6) p = 0.001
Postpartum haemorrhage	16 (29.6)	29 (53.7)	2.8 (1.2–6.1) p = 0.012	13 (24.1)	12 (24.1)	1.0 (0.4–2.4) p = 0.999	3.7 (1.6–8.3) p = 0.002
Maternal sepsis	23 (42.6)	29 (53.7)	1.6 (0.7–3.3) p = 0.249	21 (38.9)	13 (24.1)	0.5 (0.2–1.1) p = 0.100	3.7 (1.6–8.3) p = 0.002
Obstructed labour	2 (3.7)	25 (46.3)	22.4 (4.9–101.5) p<0.001	3 (5.6)	9 (16.7)	3.4 (0.9–13.3) p = 0.079	4.3 (1.8–10.5) p = 0.001
Neonatal complication	0	9 (16.7)	-	4 (7.4)	4 (7.4)	1.0 (0.2–4.2) p = 0.999	2.5 (0.7–8.7) p = 0.149
Referral systems	0	25 (46.3)	-	0	5 (9.3)	-	8.4 (2.9–24.5) p<0.001

P values are based on Z-test using logistic regression model; OR–Odds Ratio; CI–Confidence Interval; N—Denominator

### Provider preparedness

Provider knowledge and practices were assessed using staff nurse interviews and case sheet audits, respectively. Together, they give us an indication of how prepared the providers were in preventing or dealing with life-threatening complications that can emerge during institutional births. The study findings related to provider knowledge are presented in Table 3. Within both arms, significant improvements in staff nurse knowledge were observed over time in all of the parameters except those related to low birth weight definition in the intervention arm and newborn sepsis management in the control arm. However, the intervention arm performed significantly better than the control arm in 2013 with respect to knowledge of administration of AMTSL (82.4% vs. 35.8%, AOR 10, 95% C.I 5.5 to 18.2); diagnosis of eclampsia (47.8% vs. 16.8%, AOR 6.4, 95% C.I 2.9 to 13.9); use of magnesium sulphate in eclampsia (77.9% vs. 47.5%, AOR 4.8, 95% C.I 2.7 to 8.9); diagnosis of maternal sepsis (47.1% vs. 25.5%, AOR 3.3, 95% C.I 1.7 to 6.1), management of maternal sepsis (73.5% vs. 10.9%, AOR 36.1, 95% C.I 13.6 to 95.9); neonatal resuscitation (48.5% vs. 11.7%, AOR10.7, 95% C.I 4.6 to 25.0); LBW care (58.1% vs. 40.9%, AOR 2.4; 95% C.I 1.2 to 4.7); and newborn sepsis management (44.9% vs. 3.6%, AOR 46.0, 95% C.I 12.3 to 173.8). The difference in difference analysis showed significant changes in knowledge of most of the parameters except those related to maternal sepsis and low birth weight.

**Table 3:** Staff nurses’ knowledge about diagnosis and management of complications in the intervention and control facilities.

Knowledge parameter	Intervention 2012 (#/%) N = 147	Intervention 2013 (#/%) N = 136	Intervention 2012 vs.2013 AOR, C.I and p values	Control 2012 (#/%) N = 148	Control 2013 (#/%) N = 137	Control 2012 vs. 2013 AOR, C.I and p values	Intervention vs. control 2013 AOR, C.I and p values
Know all 3 steps of AMTSL (active management of third stage of labour)	9 (6.4)	112 (82.4)	84.9 (36–200.4) p<0.001	14 (9.5)	49 (35.8)	5.3 (2.7–10.4) p<0.001	10.0 (5.5–18.2) p<0.001
Know all 3 signs for diagnosis of eclampsia	2 (1.4)	65 (47.8)	96.3 (20.2–459.1) p<0.001	3 (2.0)	23 (16.8)	10.3 (2.9–36.9) P<0.001	6.4 (2.9–13.9) p<0.001
Know correct dose of magnesium sulphate for eclampsia management	32 (21.8)	106 (77.9)	13.4 (7.0–25.4) p<0.001	23 (15.5)	65 (47.5)	4.6 (2.5–8.4) p<0.001	4.8 (2.7–8.9) p<0.001
Know 3 main signs for diagnosis of sepsis	5 (3.4)	64 (47.1)	28.3 (10.0–80.0) p<0.001	2 (1.4)	35 (25.5)	28.2 (6.3–126.7) p<0.001	3.3 (1.7–6.1) p<0.001
Know all 3 drugs for sepsis management	3 (2.0)	100 (73.5)	177.4 (42.8–734.4) p<0.001	1 (0.7)	15 (10.9)	15.8 (2.0–123.4) p = 0.009	36.1 (13.6–95.9) p<0.001
Know all 4 aspects of neonatal resuscitation	3 (2.0)	66 (48.5)	66.3 (16.5–266.7) p<0.001	4 (2.7)	16 (11.7)	4.7 (1.4–15.1) p = 0.010	10.7 (4.6–25.0) p<0.001
Know correct definition of low birth weight	117 (79.6)	119 (87.5)	1.7 (0.9–3.5) p = 0.124	102 (68.9)	115 (83.9)	2.3 (1.2–4.3) p = 0.012	1.4 (0.7–3.1) p = 0.367
Know 3 important aspects of low birth weight care	23 (15.7)	79 (58.1)	10.1 (5.2–19.7) p<0.001	12 (9.1)	56 (40.9)	11.2 (5.0–25.0) p<0.001	2.4 (1.2–4.7) p = 0.010
Know 2 drugs for newborn sepsis management	4 (2.7)	61 (44.9)	57.4 (15.6–211.1) p<0.001	7 (4.7)	5 (3.6)	0.7 (0.2–2.5) p = 0.577	46.0 (12.2–173.8) p<0.001

P values are based on Z-test using multilevel logistic regression model; OR–Odds Ratio; CI–Confidence Interval; N—Denominator

The case sheet review findings are presented in Table 4. Case sheets were used for 80.2% (739/888) and 57.3% (339/647) of women in labour at the intervention and control sites, respectively. The gaps in coverage were due to staff turn-over and staff vacancies. The parameters particular to each specific category (initial assessment, labour monitoring, delivery and postpartum care) were grouped together and compared between the intervention and control arms. The staff nurses in the intervention arm were more likely to start a case sheet for each client and for those case sheets started, there was consistently greater compliance shown with the protocols than in the control arm, as seen by completed documentation during initial assessment (80% vs. 61.4%, AOR 3.6, 95% C.I 1.7 to 7.6); labour monitoring using a partograph (77.3% vs. 32.1%, AOR 25.8, 95% C.I 9.6 to 69.4); delivery and immediate post-partum care for mothers (78.6% vs. 31.8%, AOR 22.1, 95% C.I 8.0 to 61.4) and newborns (73.9% vs. 32.8%, AOR 24.1, 95% C.I 8.1 to 72.0); and newborn vaccinations before discharge (49.6% vs. 35.7%, AOR 4.7, 95% C.I 1.2 to 18.3).

**Table 4:** Case sheet audit findings in the intervention and control facilities.

#	Case sheet parameter	Intervention 2013		Control 2013		Intervention vs. Control AOR., C.I and p values
		Completed (#/%)	N	Completed (#/%)	N	
**1**	Case sheets where the documentation of initial assessment was complete (n = case sheets that indicated assessment of blood pressure, foetal heart rate, cervical dilatation and status of membranes; (N = all women > 20 weeks walking in labour or with complications))	592 (80.1)	739	208 (61.4)	339	3.6 (1.7–7.6) p = 0.001
**2**	Case sheets where partograph documentation was complete (n = case sheets that indicated time of partograph filling, cervical dilatation, contractions, maternal vitals and foetal heart rate; N = Case sheets of women who completed delivery in the facility)	545 (77.3)	705	108 (32.1)	336	25.8 (9.6–69.4) p<0.001
**3**	Case sheets where delivery and immediate postpartum period related documentation was complete for women (n = case sheets that indicated administration of AMTSL, estimation of blood loss and monitoring of temperature, pulse, blood pressure & lochia; N = Case sheets of women who stayed through the delivery and postpartum period in the PHC)	528 (78.6)	672	101 (31.8)	318	22.1 (8.0–61.4) p<0.001
**4**	Case sheets where newborn related documentation was complete during delivery and immediate postpartum periods (n = case sheets that indicated birth weight, vitamin K, breastfeeding within first hour, monitoring newborn feeding & newborn colour; N = Case sheets of newborns that stayed through the postpartum period in the PHC)	497 (73.9)	673	104 (32.8)	317	24.1 (8.1–72.0) p<0.001
**5**	Case sheets where newborn vaccination related documentation was complete (n = case sheets that indicated administration of 0 dose BCG, OPV and Hepatitis B; N = Case sheets of all newborns that were discharged healthy)	330 (49.6)	666	112 (35.7)	314	4.7 (1.2–18.3) p = 0.024

P values are based on Z-test using multilevel logistic regression model; AOR–Adjusted Odds Ratio;, CI–Confidence Interval; n–Numerator; N–Denominator

### Costing details

The total cost of developing and implementing the intervention program across the eight high priority districts in northern Karnataka over one year amounted to USD $467,371. The start-up costs (for capital expenditure and for conducting induction training for mentors and district staff) formed 12% of the total costs (USD $53,759). The annual costs, including expenditure for staff salaries and travel, communication and printing, and events such as refresher trainings, clinical postings and review meetings, amounted to USD $413,542. When the costs are computed for a single district (with an average population of 2 million), this amounts to USD $58,413 per district. When the costs are computed specifically for the PHC share of delivery load in the region, this works out to an average of $5.60 per delivery [21].

## Discussion

The onsite nurse mentoring intervention resulted in improved quality of institutional births as indicated by improvements in facility readiness and provider preparedness in the primary health centers of northern Karnataka. In recent times, there have been several attempts to develop and test models for improving the quality of maternal and newborn care, both nationally and globally [22–24], and our study establishes new evidence around an innovative and relatively low-cost model of quality improvement. This model, of periodic health worker mentoring visits with the use of a simple but comprehensive case sheet, can be scaled up in remote PHCs that serve a large rural population. The study covered 100% of facilities and close to 90% of staff nurses in two high priority districts of the region; the coverage achieved by the study helps to establish evidence for the feasibility of a district wide implementation model for quality improvement.

The program resulted in significant improvements in the availability of critical drugs and supplies in the intervention PHCs. It is already well understood that the quality of health care is significantly influenced by the availability of drugs and key clinic supplies [25,26]. Gaps in readiness of PHCs have been a concern in the region [5], and the ability of the intervention program to address these gaps offers important lessons for those working in the area of quality improvement. The nurse mentors engaged PHC staff in using self-assessment checklists periodically to assess the availability of drugs and supplies; they later developed action plans to address identified gaps with the help of untied funds, i.e. the flexible fund available in the facilities to meet emergency needs. The acceptance and use of the self-assessment process and tools appear to have influenced staff to address system readiness within the PHCs on their own initiative, as noted in other qualitative studies in the area [27].

The readiness of facilities to deal with obstructed labour and gestational hypertension improved more quickly than for the other maternal complications. This is possibly because of changes in availability of partographs with regard to obstructed labour, as well as magnesium sulphate and calcium gluconate with regard to gestational hypertension (S1 File). Facility readiness for handling newborn complications was slower to improve, although within the intervention arm the number of facilities that offered comprehensive newborn complication care went up from none to nine during the study period; this was due largely by improvements in availability and use of vitamin K and neonatal bag and mask. The suboptimal availability of emergency neonatal care in the region has already been documented, and warrants a larger systems level intervention to ensure adequate coverage and quality of neonatal care [19].

Although we observed some improvements in the availability of *routinely* used drugs (for example oxytocin and vitamin K) in both study arms, likely due to renewed government efforts at supply of basic drugs over the period of the study, the availability of *emergency* drugs and supplies (for example, magnesium sulphate, calcium gluconate, betamethasone, urine dipsticks, newborn bag and mask), improved only in the intervention facilities. This highlights the important role that the mentors played in improving staff emergency preparedness through multiple modalities, including knowledge sharing, encouragement and use of case sheets and self-assessments. It is also possible that the staff who received mentoring became more knowledgeable and skilled over time, and hence more confident in managing complications, rather than merely referring without first-line management, as evidenced by the knowledge assessments and greater use of delivery records and complication case sheets in intervention facilities. Similar observations have been made in other studies where improved awareness and confidence of managing complications led to higher medicine usage and availability [22].

The intervention program also effectively improved provider preparedness in preventing and dealing with complications that are common in the region. The mentors used multiple methods and approaches during their visits, such as using the comprehensive case sheets as a teaching aid, using models for skill training, undertaking small group teaching, and mentoring during actual patient encounters [27]. A Cochrane review concluded that educational outreach visits (EOVs), when coupled with other approaches, can influence professional health practice and outcomes [28]. Our study findings provide additional evidence to support these conclusions. There is also a growing body of evidence that contextual teaching in a reflective and dialogue based approach, can over time influence the thinking process in clinical practice [29]. The onsite visits offered opportunities for mentors to address context-specific needs in small groups over a period of time. The mentors used a teaching plan for each visit that allowed them to cover a variety of topics in a phased manner over the intervention period. This seemed to be more effective in improving staff knowledge than training that delivers all knowledge at one time, such as the current SBA training, which lasts 21 days, includes large numbers of trainees (close to 30 per batch), and requires staff to leave their facilities and families for the duration of the training, all of which may negatively affect learning. Similar observations were made in Uttar Pradesh, another large state in India, where phased capsular trainings proved to be more effective than the one-time training programs that are currently provided by the government [30]. In addition to this, the improvements in provider knowledge in this study may also have been influenced by the new project case sheet that had an integrated checklist [27]. Similar observations have been made in other parts of the region, where the use of the WHO safe birth checklist improved essential child birth practices [20]. The project case sheet is not only a comprehensive checklist for the management of routine labour, but also has supplementary management protocols for complications that arise. Thus it not only reminds staff of what is normal practice, but provides clear guidance on the differential diagnosis of complications and of what should be the pre-referral management. It thus serves as a valuable job aid to remind staff as to how to adhere to standard clinical protocols. In our study, we observed higher rates of case sheet use in the intervention facilities that received mentoring support, suggesting that the case sheet as a stand-alone checklist, without mentoring, may not be used as effectively. Poor documentation practices in the region have already been recognized [5], and providing a user friendly case sheet to the providers, complemented by constant feedback and reinforcement during mentoring visits have changed the scenario [27]. The gaps in the use of case sheets in the intervention arm points to the need for ongoing mentoring support at the facilities and periodic reinforcement by the higher health systems until coverage is adequate and sustained.

The need for implementation research that examines the effectiveness of quality improvement interventions has been much emphasized [31]. The strengths of the current study are that the new model was tested at a large operational scale, using a randomized control trial design, and that a wide range of characteristics related to quality (both at the provider and facility levels) were examined. Importantly, we were also able to demonstrate significant improvements in facility preparedness without any external injection of funds for drugs or equipment. Our study also has some limitations. Direct observations of provider practices would have helped us better to understand provider preparedness, but this was not logistically feasible in the context of the low delivery volumes experience by most PHCs. Instead, we used case sheet audits to interpret documentation of provider practices. While documentation may not always be in alignment with provider practice, we believe that the sustained support by mentors to the staff over a period of time has resulted in more comprehensive documentation, reflecting actual practice. Another limitation is that we could not ascertain the baseline status of case sheet use, as it took some time for the new case sheets to be adopted in the facilities. Before the start of this project, there was already a government-developed patient case sheet. However, we were unable to assess the use of this tool, as the case sheets were rarely used due to poor supply, absence of a culture of documentation, and their poor design and format [5]. We also experienced challenges in analysing complication case sheets due to their poor uptake in the control arm. This study establishes evidence around facility readiness and provider preparedness, but not around outcomes related to mortality. We recognize that factors outside of the facility (e.g. delays in seeking care, availability of transport, etc.) have an influence on outcomes, and this was outside the scope of the present study.

While we recognize the challenges in designing and implementing robust experimental studies in settings and at a scale encountered in a country like India, we believe that these evaluations are important, as they establish first-hand evidence about program effectiveness. At the same time, it is critical to understand the actual processes that led to the changes in the outcomes, so as to enable program managers to successfully replicate such interventions in their own settings. Recognizing this, we have documented the process in great detail, including the monitoring mechanisms that we adopted for the intervention to inform the program on a day-to-day basis. This has been prepared as a separate report and a publication [27,32].

Cost analysis also provides useful insights to program managers with respect to the budgetary implications for replication of our program model at a district or a regional level. Considering the scale of the intervention and its effects on quality of care, the cost as indicated above seems reasonable.

## Conclusion

Improving the quality of care, alongside increasing the coverage of institutional births, is key to achieving better maternal and newborn health outcomes in India. We were able to demonstrate that our onsite nurse mentoring, together with the use of simplified case sheets, improved provider preparedness and facility readiness to manage normal labour and delivery, as well as the complications at scale. We were also able to demonstrate that this program was not very costly, and so should be replicable in other contexts in India and elsewhere.

## Supporting Information

S1 CONSORT Checklist Consort Extension checklist for Cluster Randomized Trials. (DOCX)

S1 File Table showing facilities equipped with required drugs, supplies and referral systems to manage maternal and newborn complications. (DOCX)

S2 File Data file of Table 1 facility data. (DTA)

S3 File Data file of Table 1 staff nurse data. (DTA)

S4 File Data file of Table 2 data. (DTA)

S5 File Data file of Table 3 data. (DTA)

S1 Protocol Protocol for Monitoring and Evaluation of Intervention. (PDF)
