# Supplementary material for: Effectiveness of Onsite Nurse Mentoring in Improving Quality of Institutional Births in the Primary Health Centres of High Priority Districts of Karnataka, South India: A Cluster Randomized Trial
Source: PLoS One. 2016 Sep 22;11(9):e0161957. doi: 10.1371/journal.pone.0161957 (PMC5033379; doi:10.1371/journal.pone.0161957)
Supplement: S1 File — (DOCX) [file pone.0161957.s002.docx]

**S1 Table: Facilities equipped with required drugs, supplies and referral systems to manage maternal and newborn complications**

| Category | Specific parameter (drug, equipment) | Intervention 2012 (#/%)  N=54 | Intervention 2013 (#/%) N=54 | Intervention 2012 vs. 2013 OR, 95% CI and p value | Control 2012 (#/%) N=54 | Control 2013 (#/%) N=54 | Control 2012 vs. 2013  OR, 95% CI and p value | Intervention vs. control 2013 OR, 95% CI and p value |
| --- | --- | --- | --- | --- | --- | --- | --- | --- |
| Gestational hypertension | Urine dipstick | 28 (51.9) | 41 (75.9) | 2.9 (1.3-6.7)  p=0.010 | 30 (55.6) | 34 (63.0) | 1.4 (0.6-2.9) p=0.434 | 1.9 (0.8-4.3)  p=0.146 |
|  | Inj Mag sulphate | 24 (44.4) | 41 (75.9) | 3.9  (1.7-8.9) p=0.001 | 21 (38.9) | 24 (44.4) | 1.3 (0.6-2.7)  p=0.558 | 3.9 (1.7-9.0) p=0.001 |
|  | Tab Nifedipine | 31 (57.4) | 41 (75.9) | 2.3  (1.0-5.3) p=0.043 | 29 (53.7) | 27 (50.0) | 0.9 (0.4-1.8)  p=0.700 | 3.1 (1.4-7.2) p=0.006 |
|  | Inj Calcium gluconate | 2 (3.7) | 34 (63.0) | 44.2  (9.7-201.4) p<0.001 | 1 (1.9) | 21 (38.9) | 33.7 (4.3-262.7) p=0.001 | 2.7 (1.2-5.8) p=0.013 |
|  | IV fluids | 47 (87.0) | 52 (96.3) | 3.9  (0.8-19.6)  p=0.101 | 38 (70.4) | 43 (79.6) | 1.6 (0.7-4.0) p=0.269 | 6.7 (1.4-31.6) p=0.017 |
|  | Inj Betamethasone | 16 (29.6) | 43 (79.6) | 9.3  (3.8-22.5)  p<0.001 | 31 (57.4) | 33 (61.1) | 1.2 (0.5-2.5) p=0.695 | 2.5 (1.1-5.9) p=0.038 |
|  | **ALL OF ABOVE** | 0 | 19 (35.2) | - | 0 | 3 (5.6) | - | 9.2 (2.5-33.6) p=0.001 |
| Post-partum haemorrhage | Lab facility for test blood group typing | 27 (50.0) | 46 (85.2) | 5.8 (2.3-14.4) p<0.001 | 27 (50.0) | 44 (81.5) | 4.4 (1.8-10.5) p=0.001 | 1.3 (0.5-3.6)  p=0.606 |
|  | Lab facility for Haemoglobin testing | 47 (87.0) | 51 (94.4) | 2.5 (0.6-10.4) p=0.196 | 46 (85.2) | 48 (88.9) | 1.4 (0.4-4.3)  p=0.568 | 2.1 (0.5-9.0)  p=0.305 |
|  | Inj Oxytocin | 41 (75.9) | 52 (96.3) | 8.2 (1.8-38.6) p=0.007 | 39 (72.2) | 52 (96.3) | 10.0 (2.2-46.3) p=0.003 | 1 (0.1-7.4)  P=0.999 |
|  | Inj Methyler-  Gometrine | 41 (75.9) | 34 (63.0) | 0.5 (0.2-1.2) p=0.146 | 34 (63.0) | 14 (25.9) | 0.2 (0.1-0.5) p<0.001 | 4.9 (2.1-11.0)  p<0.001 |
|  | **ALL OF ABOVE** | 16 (29.6) | 29 (53.7) | 2.8 (1.2-6.1) p=0.012 | 13 (24.1) | 13 (24.1) | 1.0 (0.4-2.4)  p=0.999 | 3.7 (1.6-8.3)  p=0.002 |
| Obstructed labour | Partographs available | 18 (33.3) | 50 (92.6) | 25.0 (7.8-80.2) p<0.001 | 12 (22.2) | 31 (57.4) | 4.7 (2.0-10.9)  p<0.001 | 9.3 (2.9-29.4)  p<0.001 |
|  | Antibiotics (ampicilllin, gentamycin and metronidazole) | 27 (50.0) | 31 (57.4) | 1.3 (0.6-2.9)  p=0.441 | 25 (46.3) | 19 (35.2) | 0.6 (0.3-1.4)  p=0.241 | 2.5 (1.1-5.4)  p=0.022 |
|  | Inj Betamethasone | 16 (29.6) | 43 (79.6) | 9.3 (3.8-22.5) p<0.001 | 31 (57.4) | 33 (61.1) | 1.2 (0.5-2.5)  p=0.695 | 2.5 (1.0-5.9)  p=0.038 |
|  | **ALL OF ABOVE** | 2 (3.7) | 25 (46.3) | 22.4 (4.9-101.5)  p<0.001 | 3 (5.6) | 9 (16.7) | 3.4 (0.9-13.3)  p=0.079 | 4.3 (1.8-10.5)  p=0.001 |
| Maternal sepsis | Inj Ampicillin | 32 (59.3) | 36 (66.7) | 1.4 (0.6-3.0) p=0.426 | 32 (59.3) | 24 (44.4) | 0.6 (0.3-1.2)  p=0.125 | 2.5 (1.1-5.5)  p=0.021 |
|  | Inj Gentamicin | 47 (87.0) | 50 (92.6) | 1.9 (0.5-6.8)  p=0.346 | 51 (94.4) | 50 (92.6) | 0.7 (0.2-3.5)  p=0.697 | 1.0 (0.2-4.2)  p=0.999 |
|  | Inj Metronidazole | 45 (83.3) | 46 (85.2) | 1.0 (0.4-3.2)  p=0.792 | 47 (87.0) | 41 (75.9) | 0.5 (0.2-1.3)  p=0.142 | 1.8 (0.7-4.8)  p=0.228 |
|  | Tab Paracetamol | 52 (96.3) | 54 (100.0) | - | 54 (100.0) | 54 (100.0) | - | - |
|  | IV fluids | 47 (87.0) | 52 (96.3) | 3.9 (0.8-19.6)  p=0.101 | 38 (70.4) | 43 (79.6) | 1.6 (0.7-4.0)  p=0.269 | 6.7 (1.4-31.6)  p=0.017 |
|  | **ALL OF ABOVE** | 23 (42.6) | 29 (53.7) | 1.6 (0.7-3.3)  p=0.249 | 21 (38.9) | 13 (24.1) | 0.5 (0.2-1.1)  p=0.100 | 3.7 (1.6-8.3)  p=0.002 |
| Newborn complications (birth asphyxia, prematurity/ low birth weight and sepsis) | Antibiotics (Ampicilin and Gentamicin) | 30 (55.6) | 34 (63.0) | 1.4 (0.6-2.9)  p=0.434 | 31 (57.4) | 23 (42.6) | 0.6 (0.3-1.1)  p=0.125 | 2.3 (1.1-5.0)  p=0.035 |
|  | Inj Vitamin K | 2 (3.7) | 48 (88.9) | 208 (40.0-1080.5) p<0.001 | 7 (13.0) | 33 (61.1) | 10.6 (4.0-27.7) p<0.001 | 5.1 (1.9-14.0) p=0.002 |
|  | Oxygen cylinder and masks | 30 (55.6) | 36 (66.7) | 1.6 (0.7-3.5) p=0.238 | 32 (59.3) | 20 (37.0) | 0.4 (0.2-0.9)  p=0.022 | 3.4 (1.5-7.5)  p=0.002 |
|  | Neonatal bag with small mask | 16 (29.6) | 27 (50.0) | 2.4 (1.1-5.2) p=0.032 | 25 (46.3) | 16 (29.6) | 0.5 (0.2-1.1)  p=0.076 | 2.4 (1.1-5.2)  p=0.032 |
|  | Neonatal bag with normal mask | 40 (74.1) | 48 (88.9) | 2.8 (1.0-7.9) p=0.053 | 46 (85.2) | 42 (77.8) | 0.6 (0.2-1.6) p=0.324 | 2.3 (0.8-6.6) p=0.128 |
|  | Radiant warmer | 44 (81.5) | 44 (81.5) | 1.0 (0.4-2.6) p=0.999 | 51 (94.4) | 51 (94.4) | 1.0 (0.2-5.2) p=0.999 | 0.3 (0.1-1.0) p=0.050 |
|  | **ALL OF ABOVE** | 0 | 9 (16.7) | - | 4 (7.4) | 4 (7.4) | 1.0 (0.2-4.2)  p=0.999 | 2.5 (0.7-8.7)  p=0.149 |
| Referral systems for further management of complications | Facility maintains register for documenting referrals | 24 (44.4) | 42 (77.8) | 4.4 (1.8-10.1) p=0.001 | 28 (51.8) | 33 (61.1) | 1.5 (0.7-3.1) p=0.333 | 2.2 (1.0.-5.2)  p=0.063 |
|  | Referral directory of higher referral centers displayed | 1 (1.9) | 33 (61.1) | 83.2 (10.7-648.6) p<0.001 | 0 | 5 (9.3) | - | 15.4 (5.3-44.9)  p<0.001 |
|  | Facility has system for recording outcome of referral | 4 (7.4) | 33 (61.1) | 19.6 (6.2-62.4) p<0.001 | 10 (18.5) | 21 (38.9) | 2.8 (1.1-6.7)  p=0.022 | 2.5 (1.1-5.3)  p=0.022 |
|  | **ALL OF ABOVE** | 0 | 25 (46.3) | - | 0 | 5 (9.3) | - | 8.4 (2.9-24.5)  p<0.001 |

P values are based on Z-test using logistic regression model; OR – Odds Ratio; CI – Confidence Interval; N - Denominator
