# Supplementary material for: Effectiveness of Onsite Nurse Mentoring in Improving Quality of Institutional Births in the Primary Health Centres of High Priority Districts of Karnataka, South India: A Cluster Randomized Trial
Source: PLoS One. 2016 Sep 22;11(9):e0161957. doi: 10.1371/journal.pone.0161957 (PMC5033379; doi:10.1371/journal.pone.0161957)
Supplement: S1 Protocol — (PDF) [file pone.0161957.s007.pdf]

Monitoring and evaluation of the MNCH mentoring (MM) intervention in two districts of Karnataka

Project background

In alignment with the NRHM's (National Rural Health Mission) objectives and approaches, the Bill & Melinda Gates Foundation's (the Foundation's) Maternal and Neonatal Health (MNH) Strategy seeks to improve MNCH outcomes in the world's poorest regions by catalyzing health system responses to ensure that critical, proven interventions during pregnancy and in the neonatal period reach underserved populations. While the NRHM provides a broad canvas with processes and funding mechanisms to achieve health goals, the Foundation's strategy helps in focusing on a critical technical intervention package and solution levers to enhance the performance of health systems. The Foundation has awarded funds to the University of Manitoba/Karnataka Health Promotion Trust to support the Government of Karnataka (GoK) to develop and implement strategies to improve maternal, newborn and child health in alignment with the NRHM's objectives and approaches. This is the *Sukshema* project. The project focuses on improving the availability, accessibility, quality, utilization and coverage of critical MNCH interventions among the rural poor in eight priority districts in northern Karnataka: Bagalkot, Bellary, Bidar, Bijapur, Gulbarga, Koppal, Raichur and Yadgir.

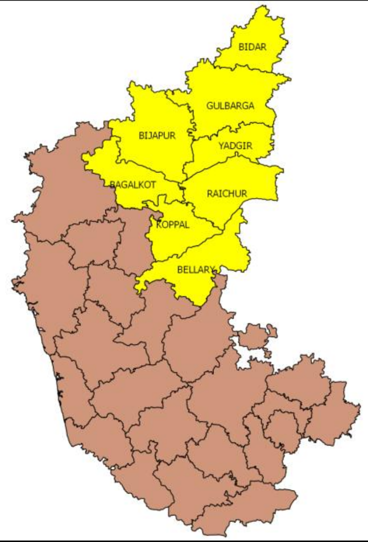

The **goal** of the project is to support the state of Karnataka and India to improve maternal, newborn and child health outcomes in rural populations through the development and adoption of effective operational and health system approaches within the NRHM. To achieve this goal, the project is designed to integrate and align key aspects of the Foundation's MNH strategy with the NRHM's health system infrastructure and mechanisms in the eight project districts, with the following four key objectives.

1. Enable expanded **availability** and **accessibility** of critical MNCH interventions for rural populations.
2. Enable improvement in the **quality** of MNCH services for rural populations.
3. Enable expanded **utilization** and population **coverage** of critical MNCH services for rural populations.
4. Facilitate identification and consistent **adoption** of best practices and innovations arising from the project at the state and national levels.

As part of the effort to improve the quality of MNCH services, the Sukshema Project will pilot and evaluate a model of on-site MNCH mentoring (MM) in 24/7 PHCs in two districts.

The on-site MNCH mentoring (MM) for improved clinical care and service delivery intervention

Intervention background and rationale

In the PHCs (primary health care centres) in the Sukshema project districts, only 63% of staff nurses have been trained in skilled birth attendance (SBA) and 12% of medical officers have been trained in BEmONC (Basic Emergency Obstetric and Newborn Care). In addition, there are no mechanisms after training for follow-up of staff to ensure good clinical practice and maintenance of skills. While basic training may provide the knowledge required, it often does not provide the confidence to translate the knowledge into practice, or there may be service delivery issues that hamper staff ability to practice good quality care. Service provider assessments conducted by the Sukshema project suggest that knowledge levels are low, and actual skills and abilities are even lower. In an assessment of intranatal care knowledge, less than 70% of staff nurses knew that AMSTL (active management of third stage of labour) was essential for all deliveries and only 28% knew the proper steps in AMSTL. For postnatal care, providers scored only 52% on knowledge questions, and on observation, their practice was correct in just 31% of provider/client interactions. Only 13% of providers were able to perform all the components of a postpartum physical exam. Clinical mentoring can complement in-service training to build competencies and confidence of providers on-site – that is, in the actual context where they are providing care.

However, clinical competency alone is not sufficient to ensure quality service delivery. The delivery of quality services is influenced by such things as staffing patterns and schedules, infection prevention practices, referral practices, equipment and supply logistics, record keeping, lack of a supportive environment for staff, lack of teamwork, and lack of staff attention to patient rights to information, respect and dignity. Of particular concern is weak referral processes and follow-through once referrals are made, which compromise providing a continuum of care for mothers and newborns. Attention to these aspects of service delivery requires that all staff at the PHC be involved in developing a common understanding of what it means to provide a quality service, their roles in providing these services across the continuum of care, and how to support each other and problem-solve to improve services.

Project Sukshema proposes to introduce an innovative strategy of providing on-site clinical mentoring and supportive supervision to staff nurses in PHCs (year 1 focus) through a dedicated cadre of nurse mentors. The aim is to develop and pilot a replicable model of on-site mentoring to ensure clinical competencies, strengthen referral processes and improve MNCH service quality.

Hypothesis

By providing on-site mentoring for improved clinical care and service delivery, we hypothesize that the quality of services and care will improve and patients will have better clinical outcomes. Specifically we believe that routine on-site clinical and service delivery mentoring will strengthen clinical knowledge, skills and practices among staff nurses, will strengthen appropriate referrals and follow up care, will improve other aspects of service quality, and provider and client satisfaction. The evaluation will assess change *during the first year* of the intervention that focuses on clinical mentoring of staff nurses and on quality improvement in the PHCs as a whole. The evaluation will focus on success indicators as shown in Appendix 1. Due to the short time frame for evaluation, success indicators will be principally at the levels of inputs, outputs, and outcomes focusing on adherence to the Sukshema Critical Care package, although we also hope to assess some key health outcomes, in terms of reduction of “near-miss” cases.

Intervention overview

Geography and site selection

Using a cluster randomized control study design, the MM (MNCH mentoring) intervention will be evaluated in PHCs in two districts, Gulbarga and Bellary that have a total of 122 24x7 PHCs (Gulbarga 70; Bellary 52). Sixty one PHCs (Gulbarga 35; Bellary 26) will be randomly assigned to an MM intervention or experimental group (stratified by taluka and 6-monthly patient delivery load), while the rest will serve as control sites.

Pre-baseline (SBA+) training

The first part of the intervention involves *all* the 122 PHC 24/7 facilities in the two districts for two reasons: first we feel that this training is generally needed and will help to put the intervention and control site staff on the same level playing field in terms of basic MNCH knowledge; second, we know from the Sukshema baseline survey conducted in 2011 that few staff use adequate reporting mechanisms such as case sheets. As these are needed both programmatically as job aids, and for programme evaluative purposes, we will use this training as an opportunity to introduce new case sheets. After the first phase of baseline survey (described below) and before the mentoring programme begins in the intervention sites, Sukshema project staff will conduct a 3 day training update (called SBA+) for all staff nurses and a one day SBA+ for all M.O.'s from all 122 PHCs in the two districts, irrespective of whether they have previously attended the NRHM Skilled Birth Attendant (SBA) training. The 3 day training will focus on the essential components on intra-partum and postpartum (mother and baby) care identified in the Sukshema critical care package that evidence shows can significantly reduce maternal and newborn morbidity and mortality. The one day training for doctors will focus on identification of danger signs, pre-referral management of complications, quality of care, case audits and improvement of management and documentation. At these trainings, staff will be given, and instructed in the use of enhanced case sheets for women and babies from labour through to discharge, including use of the partograph. After this training, there will be no further interventions in the control sites, except that Sukshema staff will visit every 3 months to collect copies of the case sheets.

MNCH Mentoring

In each district MNCH mentors (MMs), a new staff cadre, will be recruited and trained (Gulbarga 5; Bellary 4), each responsible for the intervention in 6-7 *experimental/intervention* PHCs. We will also train 3 additional MMs to act as a back-up (2 in Gulbarga and one in Bellary), total 12 mentors. The MMs will attend some of the PHC staff training but will also be trained for 4 weeks by project staff at St John's Medical College in essential clinical competencies and in how to mentor staff in clinical skills, team building and problem solving, service delivery improvement, as well as in how to be effective mentors. The MNCH mentors will be senior nurses, selected based on specific criteria such as clinical experience, training and mentoring abilities, and experience/familiarity with government health systems. They will be guided by a Mentoring Coordinator in each district who will support the mentors' activities and liaise between them, the PHCs, district government staff and KHPT district staff. During the first year, the mentors will be trained by the Project to (a) provide clinical mentoring in safe delivery to staff nurses by hands-on coaching; (b) provide mentoring in how to improve the quality of services by building teamwork and introducing problem solving initiatives to address other aspects of service delivery (including especially referral processes); and in year 2 to mentor ANMs who will come to the PHC during the MNCH mentor visits, in providing antenatal, postpartum and newborn care, and referrals and follow up.

After the trainings, the MMs will visit each of their assigned intervention PHCs for 2-3 days every 2 months during the first year of the programme. The mentors will use tools and approaches such as observations, clinical audits, and interviews as aids to make an assessment of capacities in the

facilities. In year one they will attempt to upgrade staff nurse skills through case reviews and audits, demonstrations and modelling of good practice, bed-side case discussions and small group teachings on such issues as partograph use, infection prevention, family planning, breastfeeding and complementary feeding, baby care, referral practices and record-keeping. Mentors will bring mannequins and other training aids to the site to provide skills practice for mentees in the event patients are not available or if additional practice is required. Although the focus of attention during the evaluation will be on the staff nurses, other staff who are involved in delivery and postpartum care will also be helped to improve patient care, for example the M.O.'s who are called to assist with complicated cases, the ward attendants who are responsible for infection prevention and other nurses who might help with postpartum care. Improvements in this area will be also be documented. Apart from clinical mentoring, the mentors will focus on problem-solving around all aspects of the provision of quality MNCH services. A team approach will be adopted to address specific problem areas of concern to staff such as (but not limited to): staffing patterns and schedules; infection prevention practices; referral practices; equipment and supply logistics; record keeping; staff support; teamwork; and staff attention to patient rights to information, respect, dignity and friendly services. Part of the quality improvement work will include review of all case sheets and log books/registers, collation of data with staff and discussion of case management, focusing especially on cases where there have been problems or complications requiring referral. Thus the intervention/experimental sites will have the full package of interventions (SBA+ training for staff nurses and doctors plus on site mentoring), whereas the control sites will merely have the short SBA+ training for staff nurses and doctors.

The State Institute of Health and Family Welfare, Government of Karnataka, has already expressed an interest in adding mentoring into existing capacity building systems and has requested the Sukshema project to demonstrate sustainable and effective mentoring systems. The project will continue to involve the state and district health officials and programme officers in the development/adaptation of manuals and curriculum, training and support for mentors, and monitoring the pilot intervention. The evaluation of the intervention will be shared with them to help in integrating this model in the remaining project districts.

Monitoring and Evaluation overview

Very little data exist on the utility of such a model and thus rigorous evaluation of this new intervention is required. Below, we outline an experimental design to answer the critical question of whether a training update plus regular on-site mentoring as described above, is more effective in improving services and achieving better patient outcomes, than merely provision of the training update. Using a cluster randomized control study design; the evaluation will be at two levels: routine on-going monitoring; and pre and post MM intervention evaluation.

The overall key objectives of the evaluation of this pilot project are:

1. To document operational details and the implementation processes, and evaluate the effectiveness of the intervention model to inform scale-up of the intervention, using 61 intervention sites and 61 matched control sites. The evaluation will focus on the list of success indicators shown in Appendix 1.
2. To accumulate data that will inform any modification of the intervention model and then institutionalize it outside of the pilot sites and districts.

A. Pre and post MM intervention evaluation strategy

Evaluation design
Experimental study design (cluster randomized control trial) will be used to evaluate the effectiveness of mentoring intervention.

Site selection and randomization
Bellary and Gulbarga districts that have a total of 122 PHCs will provide the sites for intervention. The sites will be randomized into intervention and control after the initial (SBA+) training.

Evaluation phases
The "pre-intervention" evaluation will have two phases. The first phase will take place in all 122 sites before any intervention activities begin (Figure 1). This evaluation will focus on site readiness to provide quality services, provider knowledge, attitudes and reported or observed (or simulated) practices, supervision and mentoring, record keeping, and provider and client satisfaction. The NRHM SBA guidelines, PHC 24/7 guidelines, MHFW NSSK guidelines, and the Sukshema critical care package will all be used to determine expectations of clinical quality of care at this level.

The focus of the first year of the MM intervention is enhanced clinical skills in the intra-partum and postpartum periods and thus assessments of actual clinical practices are important. However, data on actual practices in the intrapartum and postpartum periods will be difficult to collect by observation during this baseline (especially those with complications). Most of the PHCs conduct only a few deliveries each week, and it will be therefore almost impossible to obtain a representative sample of observations. Therefore we will assess intrapartum and postpartum clinical practices largely though the audit of case sheets and registers. However, these case sheets are currently either not available or not used. Thus our strategy necessitates delay of this part of the second phase of the baseline until after the update training where all staff will be given and instructed in the use of new and improved case sheets. All sites will be given the opportunity to practice using these patient case sheets for one month before they are collected by project staff and used as a basis for assessing both compliance with the new case sheets, adequacy of reporting, and actual case management. Actual case observations will be done during routine MM visits where possible. The post intervention phase of the assessment will take

place in all sites once year after the first (planned 6) MM visits and will repeat the baseline survey.

Figure 1: Intervention and evaluation strategy

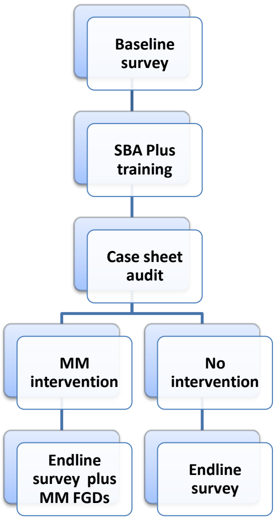

Pre and post MM intervention evaluation survey tools

The survey will use four types of survey tools pre and post MM intervention and one additional tool after one year in the intervention sites only:

A1. Questionnaires for all staff nurses on duty (including night duty) at the PHCs during the evaluation that will assess knowledge of intrapartum, postpartum, newborn care and infection prevention, and solicit provider perspectives on mentoring and supervision. In some cases, the questions will be in the form of case studies or asking for simulated demonstrations of practices.

A2. Facility audits and observations in all PHCs to assess site readiness to provide quality services and provide checks of drugs used: equipment and drugs, staffing and training levels, availability and correct use of case sheets, registers and referral slips. This tool will also have a section for observing practices in the postnatal ward.

A3. Postpartum client questionnaires with all women who have delivered up to one month before the survey traced to their homes that will assess client recall of information and services given and client satisfaction. Delivery registers will be checked and all women who delivered in the PHC n the previous month will be traced to their homes for interview.

A4. Case sheet and register audits for all cases for the one month before survey dates to assess completeness of reporting and appropriate client management

A5. Focus Group Discussions with SNs in a sample of intervention PHCs: During the post MM intervention survey we will conduct focus group discussions (FGDs) with staff at a selection of intervention sites to understand their experiences with the MM intervention and to gather their suggestions for improvement.

Data collection
The field staff will consist of three supervisors per district, plus 13 teams of 2 interviewers, one of whom will manage the facility audits and staff surveys, and one the postpartum client questionnaires for each of the 122 PHCs. It will be necessary for each team to spend 2 days in each PHC, thus each team will be able to complete the survey work in 3-4 weeks. Simultaneous data collection will be carried out in both districts.

A week's training will be provided to the field teams that will include lectures on technical topics, demonstration interviews, mock interviews and field practice. The training will be provided to about 10% more persons than actually required for the survey, and the required number will be selected based on their performance during the training. KHPT and SJMC staff will conduct the training and will monitor the field work activities and quality of data.

Informed consent
Written, witnessed informed consent will be sought from the head of the PHC who will be asked to give permission to survey the PHC; from staff nurses who will be interviewed in the PHCs and from postnatal women traced to the community

Data processing
Double data entry will be done at KHPT, who will prepare a report of the pre-intervention survey and end-line survey comparison.

Data analysis
Changes in quality of care (facility readiness and staff competencies) will be compared before and after intervention within and between the intervention and control sites. Appropriate statistical tools will be used to control for any external factors.

B. Routine continuous monitoring
First, project staff will routinely document staffing, training, financial and other project inputs, data that will be crucial for assessing the feasibility of scale up to other districts. During bi-monthly MM visits, staff will have a proscribed list of data to collect. As well as keeping details of staff at the sites their training background and any clinical mentoring given, the mentors will have a proscribed list of clinical skills to observe; wherever possible during their 3 day visits, they will observe women in labour, after delivery and in the postpartum ward, and where this is not possible, they will use models and case studies to assess staff skills. There will be continuous monitoring of the MM interventions through reports of MM visits that will include: documentation of visits through trip reports that detail facility meetings, talks given, happenings in the PHC or area and notes on any morbidity and mortality discussions; completed clinical competency checklists for mentored individuals. They will also photocopy the case sheets, which will have been used as a basis for discussion during the visit and facility action plans, for review by KHPT clinical staff. The case sheets will also be collected quarterly from the control sites by the KHPT district staff. These staff will encourage use of the case sheets at each of their quarterly visits without any additional training or mentoring.

Routine monitoring survey tools

B1. MM trip reports

B2. MM labour and newborn care observations checklists (or use of models where cases not available)
B3. Facility action plans developed during self-assessment exercises during visits

B4. Case sheet and register reviews

Key activities and timelines

Activities/Months	Dec 11 – Mar 12	April 12	May 12	Jun 12	Aug 12 – July 13		Aug 13
Study preparations – tools, ethics submission, training of field investigators	X						
Data collection (baseline, pre intervention)		X					
Training (SBA+)			X				
Case sheet audits				X			
Intervention roll out					X	X	
Routine monitoring of intervention					X	X	
Data collection (endline, post intervention)							X

Appendix 1: MNCH Mentoring Intervention Success Indicators

Inputs

- KHPT and SJMC staff time
- Training curriculae, mentoring manual , evaluation case studies and checklists developed
- New case sheets developed and supplied to 122 sites
- Mentors hired
- Mentors trained for 4 weeks and certified after training
- Mentors supported through review meetings with district officials
- Mentors re-certified after one year
- Doctors trained for 1 day in SBA+
- Staff nurses trained for 3 days in SBA+
- Funds contributed

Outputs

- CCP training for approximately 100 Drs and 300 SNs; MM training for 11 MMs
- 11 Mentors in place
- Number of visits made follows protocol for 6 bimonthly visits
- Number of review meetings with district officials
- All SN staff and doctors trained in how to use new case sheets
- All MM visits include staff problem solving meetings
- All SNs available during MM visit are provided with coaching/ mentoring on some aspect of the critical aspects

Outcomes

Outcome: Intrapartum Care: Improvement in knowledge of infection prevention

Indicator 1: % providers who know how to prevent infection in women during labour, delivery and women and newborns in the postpartum period.

Outcome: Intrapartum Care: Improvement in knowledge of labour
Indicator 2: % providers who know how to do AMTSL
Indicator 3: % providers who know how to use a partograph
Indicator 4: % providers who know what are danger signs needing referral
Indicator 5: % providers who know to identify and manage suspected pre-eclampsia and eclampsia
Indicator 6: % providers who know to identify and manage women with preterm labour
Indicator 7: % providers who know to identify and manage PPH
Indicator 8: % providers who know to identify and manage maternal sepsis
Indicator 9: % providers who know to identify and manage obstructed labour
Indicator 10: % providers who know to identify and manage fetal distress

Outcome Intrapartum Care: Improvement in provider performance during labour
Indicator 11: % labours with fully completed essential general history taking documentation
Indicator 12: % labours with fully completed history of the current labour on admission
Indicator 13: % labours with fully completed obstetric history taken on admission
Indicator 14: % labours with all appropriate exams and checks done on admission
Indicator 15: % women in labour who have high BP, who also have urine tested, on admission
Indicator 16: % labours correctly monitored by partograph
Indicator 17: % labours in which all steps of AMSTL correctly followed
Indicator 18: % women who need referral, transported within one hour of diagnosis of a complication requiring referral

Outcome immediate postpartum care in the PHC: Improvement in knowledge of PP care
Indicator 19: % providers who know what to monitor IN THE MOTHER in the immediate PP period

Outcome immediate postpartum care in the PHC: Provider performance improves in PP period
Indicator 20: % postpartum women staying in the facility for at least 12 hours and for 2 days or more
Indicator 21: % postpartum women properly monitored 2 and 4 hourly

Outcome immediate postpartum care in the PHC: clients more informed
Indicator 22: % PHC clients who were given FP counselling
Indicator 23: % PHC clients who were given breastfeeding information or counselling in ward
Indicator 24: % PHC clients who were given information about warning signs after discharge
Indicator 25: % PHC clients reporting satisfaction with all aspects of labour and postpartum care – given information, privacy, dignity, confidentiality, safe services etc

Outcome immediate newborn care: Provider knowledge improves
Indicator 26:% providers who know components of immediate neonatal care for all infants
Indicator 27: % providers who know newborn danger signs
Indicator 28: % providers who know how to initiate care for the newborn without spontaneous respiration:
Indicator 29: % providers who know to identify and manage LBW
Indicator 30: % providers who know to identify and manage newborn sepsis
Indicator 31: % providers who know about cord care
Indicator 32: % providers who know what are the key things to monitor in newborns in the immediate PP period
Indicator 33: % providers who know how to help a woman breastfeed

Indicator 34: % providers who know what information to give women/families about newborn care

Outcome immediate newborn care: Provider performance improves
Indicator 35: % babies breastfed within one hour of birth
Indicator 36: % babies immunized with BCG, Hep B and Oral Polio and Vitamin K
Indicator 37: % babies in the post natal ward who are appropriately cared for.

Outcome immediate newborn care: Clients more informed
Indicator 38: % PHC clients reporting they were told about child care
Indicator 39: % PHC clients reporting they were told about newborn warning signs

Outcome mentoring and quality of care: staff more satisfied
Indicator 40: % providers interviewed expressing various aspects of job satisfaction

Outcome mentoring and quality of care: staff feel more skilled as a result of supervision or mentoring
Indicator 41: % providers interviewed reporting being helped to improve skills

Outcome mentoring and quality of care: PHCs perform better
Indicator 42: % PHCs having action plans
Indicator 43: % PHCs having all needed drugs
Indicator 44: % PHCs having all needed equipment and supplies for delivery
Indicator 45: % PHCs having all needed equipment and supplies for infection prevention
Indicator 46: % PHCs having all needed equipment on emergency tray in labour room
Indicator 47: % PHCs that have a complete newborn corner
Indicator 48: % PHCs that have all needed IEC and learning materials
Indicator 49: % PHCs that have a list of referral facilities/displayed referral chart
Indicator 50: % PHCs that have a functioning feedback mechanism for women referred to FRU
Indicator 51 % PHCs that follow correct steps in infection control
Indicator 52: % PHCs that have an M&M audit meeting at least once per quarter with notes recorded

Outcome utilization: Client numbers increase
Indicator 53: Number of ANC visits per month in 2011 and 2012
Indicator 54: Number of women admitted in labour per month in 2011 and 2012

Outcome: Identification and management of complications
Indicator 55: % of women in labour with maternal complications and % of newborns with complications
Indicator 56: No. of labours that are obstructed (and % appropriately managed)
Indicator 57: No. of labours with PPH (and % appropriately managed)
Indicator 58: No. of labours that are pre-eclampsia cases (and % appropriately managed)
Indicator 59: No. of labours that are eclampsia cases (and % appropriately managed)
Indicator 60: No. of labours that are maternal sepsis case (and % appropriately managed)
Indicator 61: % live babies with sepsis (and % appropriately managed)
Indicator 62: % live babies with asphyxia, successfully resuscitated within one minute
Indicator 63: No live babies born LBW (and % appropriately managed)

Poor outcomes
Indicator 64: No. of stillbirths, neonatal deaths, maternal deaths
